# Glycoprotein nonmetastatic melanoma protein B (GNMPB) as a novel biomarker for cerebral adrenoleukodystrophy

**DOI:** 10.1038/s41598-022-11552-7

**Published:** 2022-05-14

**Authors:** Leyla A. Taghizadeh, Carina J. King, David R. Nascene, Ashish O. Gupta, Paul J. Orchard, LeeAnn Higgins, Todd W. Markowski, Erin E. Nolan, Justin W. Furcich, Troy C. Lund

**Affiliations:** 1 Pediatric Blood and Marrow Transplant Program, Global Pediatrics, Division of Pediatric Blood and Marrow Transplantation, MCRB, University of Minnesota, Room 460G, 425 East River Road, Minneapolis, MN 55455 USA; 2 Department of Diagnostic Radiology, University of Minnesota, Minneapolis, 55455 USA; 3 Department of Biochemistry, Molecular Biology and Biophysics, University of Minnesota, Minneapolis, 55455 USA

**Keywords:** Biomarkers, Prognostic markers, Paediatric neurological disorders

## Abstract

Adrenoleukodystrophy (ALD) is an X-linked peroxisomal disease caused by a mutation in the *ABCD1* gene, producing mutations in the very long chain fatty acid transporter, ALD protein. Cerebral ALD (cALD) is a severe phenotype of ALD with neuroinflammation and neurodegeneration. Elevated levels of Glycoprotein Nonmetastatic Melanoma Protein B (GNMPB) have been recently documented in neurodegenerative diseases such as Alzheimer’s disease, Multiple Sclerosis and Amyotrophic Lateral Sclerosis. Our objective was to measure the levels cerebral spinal fluid (CSF) GNMPB in cALD patients to determine if GNMPB could be a potential biomarker in tracking cALD disease progression. CSF GNMPB levels were significantly higher in cALD patients versus controls (2407 ± 1672 pg/mL vs. 639.5 ± 404 pg/mL, *p* = 0.0009). We found a positive correlation between CSF GNMPB and MRI disease severity score levels (R^2^ = 0.3225, *p* < 0.0001) as well as the gadolinium intensity score (*p* = 0.0204). Boys with more severe neurologic deficits also had higher levels of CSF GNMPB (*p* < 0.0001). A positive correlation was shown between CSF GNMPB and another biomarker, chitotriosidase (R^2^ = 0.2512, *p* = 0.0244). These data show that GNMPB could be a potential biomarker of cALD disease state and further studies should evaluate it as a predictor of the disease progression.

## Introduction

X-linked Adrenoleukodystrophy (ALD) is a peroxisomal disorder that results from a mutation in the *ABCD1* gene, encoding for the adrenoleukodystrophy protein (ALDP)^1^. ALDP is a peroxisomal transporter that imports very long chain fatty acids (VLCFA) into the peroxisome^2^. The mutation in the *ABCD1* gene leads to the accumulation of VLCFAs in tissues and fluids throughout the body^3^. ALD primarily affects the adrenal cortex, testis, and nervous system. Cerebral adrenoleukodystrophy (cALD) is a severe phenotype of disease which may affect up to 40% of boys with ALD and manifests as a progressive neuroinflammatory demyelinating process with onset often in childhood^4–6^. After the initial manifestations of clinical symptoms associated with cerebral inflammation, the five-year untreated survival rate is 59%^7^. Once neuroinflammation occurs, only allogeneic hematopoietic cell transplants (HCT) or gene therapy have been shown to decelerate or arrest clinical disease progression in patients where demyelination has begun^8–10^.

Newborn screening has begun in the United States and The Netherlands as early detection of adrenal insufficiency and expedient identification of cerebral changes (determined by magnetic resonance imaging) are key to the best outcome after HCT^11,12^. Despite the increase in newborn screening for ALD, there are still no robust biomarkers to predict who will develop cALD and the rate of cALD progression before or after HCT.

Glycoprotein Nonmetastatic Melanoma Protein B (GNMPB) is a multifaceted type I transmembrane protein which is also found in a secreted form from monocyte/macrophage lineage cell types but appears to have a more ubiquitous expression pattern than previously appreciated^13,14^. There has been accumulating evidence in the use of GNMPB as a biomarker across a spectrum of diseases involving the liver, kidneys, and some cancers^15,16^. Elevated levels of GNMPB have been found in neuroinflammatory and demyelinating diseases such as Multiple Sclerosis, Gaucher disease, Alzheimer’s disease, Niemann-Pick Type C disease, and Amyotrophic Lateral Sclerosis^14,17,18^. Of note, Zigdon et al. were able to show elevated CSF GNMPB in Gaucher patients, which correlated to symptom severity^19^. Genetic treatment of Gaucher in mouse models, or treatment of humans by enzyme replacement therapy (ERT), has shown corresponding improvement in elevated plasma GNMPB levels (known as osteoactivin in the mouse), providing more evidence of GNMPB as potentially useful biomarker in this disease setting^19,20^. We sought to explore the potential of GNMPB as a biomarker in ALD in a cohort of boys with cALD.

## Results

**Figure 1:**
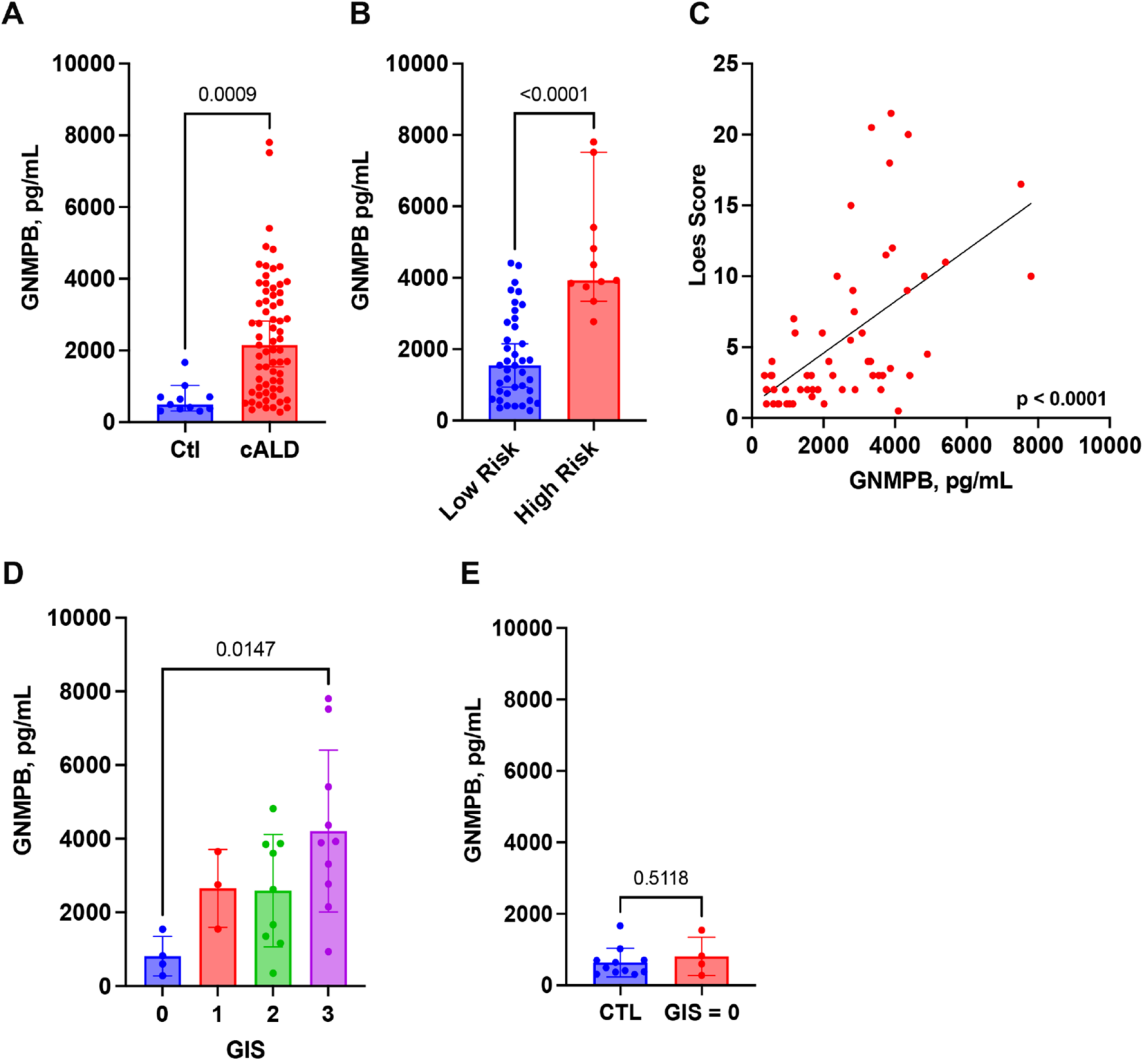
CSF GNMPB levels are elevated in boys with cALD. (**A**) shows levels of CSF GNMPB. (**B**) shows the difference between low risk and high-risk boys based on a Loes score cutoff of ≥ 10. (**C**) shows Loes score as a continuous variable and *p*-value derived from a linear regression analysis. (**D**) shows the comparison of CSF GNMPB amongst cALD patients categorized by GIS. Two-comparisons shows *p*-values derived from a Student’s t-test. *P*-value in (**D**) was derived from an ANOVA and Tukey post-hoc test. The main effect p-value was *p* = 0.0204.

To investigate GNMPB as a biomarker, CSF collected on 67 cALD boys at initial evaluation and quantified by ELISA was analyzed. The median and range for the cALD group was 2152 pg/mL and 281–7803 pg/mL, respectively. The median and range for the non-cALD group was 493 pg/mL and 312–1664 pg/mL, respectively. We found CSF GNMPB of cALD patients to be significantly greater (*p* = 0.0009) than the non-cALD patients (Fig. 1A). The Loes score is an MRI-based scoring system that quantifies the extent of cerebral disease in cALD patients based on T2-weighted lesion location and the presence of atrophy^21^. This score can be used to risk-stratify cALD patients pre HCT and can predict severity of progression^9^. A score of 10 or greater denotes the “high risk” patient group. Figure 1B shows there is a significant difference between patients in the low risk and high-risk groups (means 1761 pg/mL and 4676 pg/mL, respectively; *p* < 0.0001). We also determined that the Loes score, as a linear variable, had a correlation with CSF GNMPB levels (Fig. 1C,  R^2^ = 0.3225, *p* < 0.0001). The gadolinium intensity score (GIS) is evaluated on brain MRI after gadolinium contrast infusion and compares the intensity of the gadolinium contrast signal on T1-weighted MRI to the choroid plexus as an intra-patient reference. GIS has been shown to marker of disease severity and progression after HCT^22,23^. A subset of our cohort had a GIS measurement, and we also included 5 patients with ALD but did not have active neuroinflammatory disease (GIS = 0). Figure 1D shows that individuals with the highest levels of GNMPB also had the most elevated GIS (*p* = 0.0082). For individuals with GIS = 0, asymptomatic and without gadolinium enhancement, their GNMPB levels were different from control non-cALD controls (Fig. 1E, *p* = 0.5118) suggest a link between GNMPB and active cALD.

**Figure 2:**
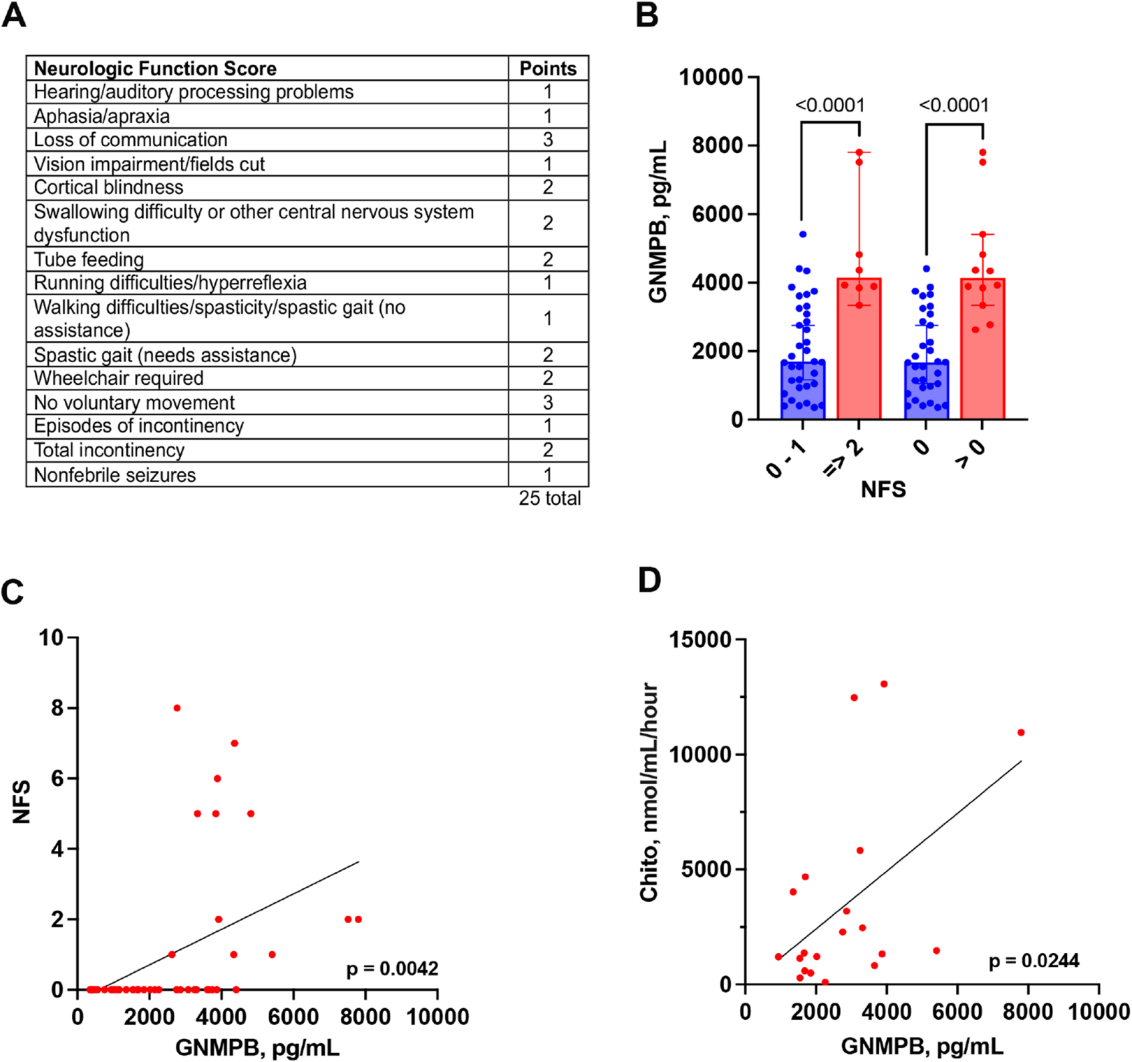
CSF GNMPB correlates with neurologic function and chitotriosidase. (**A**) The cerebral adrenoleukodystrophy neurologic function score (NFS), which was used to evaluate gross clinical neurologic severity for the cALD cohort pre-transplantation. Note that a score of zero denotes absence of clinical signs of cerebral disease. Maximal signs within a domain score the total of all grades within that domain (for example, a patient with “total urinary or fecal incontinency” scores 3, for the sum of “Episodes of incontinency” [1 point] and “Total Incontinency” [2 additional points]). (**B**) The difference between neurologically affected boys (NFS ≥ 2) versus those with NFS ≤ 1 is depicted. (**C**) Use of the NFS as a continuous variable and *p*-value derived from a linear regression analysis. (**D**) The correlation between CSF GNMBP and CSF chitotriosidase and *p*-value derived from a linear regression analysis. (**D**) shows the comparison of CSF Two-comparisons shows *p*-values derived from a Student’s t-test.

The neurologic function score (NFS) is used to evaluate the neurologic (dys)function in cALD patients, with points accumulated for loss of function in specific domains as originally reported by Moser et al.^9,24^. These results are shown in Fig. 2A. An NFS of 2 or greater has been classified as neurologically abnormal by Moser et al^24^. Figure 2B shows a significant difference between patients with NFS ≤ 1 and those with an NFS of 2 or greater (mean 2095 pg/mL versus 4939 pg/mL, *p* < 0.0001). There was also a significant difference between those with NFS = 0 (i.e. asymptomatic) and those with 1 NFS point or higher (mean 1905 pg/mL versus 4554 pg/mL, p < 0.0001). We also determined that the NFS, as a linear variable, had a correlation with CSF GNMPB levels (Fig. 2C, *p* = 0.0042), though the variance was not strong (R^2^ = 0.1794). Chitotriosidase is an enzyme released by activated monocytes/macrophages and microglia and been shown to be a CSF biomarker in Gaucher and cALD^25,26^. Figure 2D shows a positive correlation between CSF GNMPB and CSF chitotriosidase activity (R^2^ = 0.2512, *p* = 0.0244).

## Discussion

This is the first study to evaluate the use of CSF GNMPB as a novel biomarker for cALD. High levels of GNMPB correlate to the extent of disease visualized on MRIs both in terms of Loes score and GIS. A clinical reflection of higher disease burden was the loss of neurologic function, as evaluated with the NFS. We have previously shown that cALD CSF contains a number of inflammatory proteins which are highly elevated, which is not surprising given that active cALD is an inflammatory process^26–28^. Regarding GNMPB as a potential biomarker, there are remaining questions such as what the cell source is of GNMPB, and whether GNMPB is inflammatory or anti-inflammatory.

Historically, GNMPB was found to be expressed in osteocytes and melanoma cells (hence its two names: osteoactivin and Glycoprotein Nonmetastatic Melanoma Protein B)^29,30^. Recently, it is appreciated that GNMPB is more ubiquitous, and includes expression by microglia and neurons. The brains of patients with neurodegenerative conditions have been shown to have higher levels of GNMPB. For example, the brains from patients with Alzheimer’s disease (AD) have intense GNMPB staining in microglial cells^31^. Similarly, GNMPB levels were also shown to be increased in the substantia nigra of post-mortem brain samples from Parkinson’s disease (PD) patients^32^. Like many other CSF biomarkers, GNMBP may not be disease specific. For example, the degree of elevation of CSF GNMPB levels failed to separate AD patients from those with other neurologic conditions (schizophrenia, normal pressure hydrocephalus, depressive disorders or addictive disorders and dementia^33^.

There are several lines of evidence that GNMPB has an neuroprotective or anti-inflammatory role. Budge et al. showed that in an MPTP-induced mouse model of PD, that transgenic overexpression of GNMPB reduced gliosis and prevented microglial morphological changes^34^. Additionally, recombinant GNMPB attenuated LPS-induced inflammation in primary mouse microglia^34^. GNMPB has neuroprotective effects in models of ALS, with Nagahara et al. showing that recombinant GNMPB ameliorated motor neuron cell death induced by transfection of mutant TDP-43, a transactive response DNA binding protein used to model ALS^35^. Interestingly, we found that chitotriosidase correlated with GNMPB expression levels, but unlike GNMPB, chitotriosidase still remains a largely a marker of inflammation in neurodegenerative disease such ALS, AD, Gaucher, and cALD as it is thought to be released upon monocyte/macrophage or microglial cell activation^25,28,36,37^. Though recently, two studies indicate its potential to rescue neuropathy in D-galactose (D-gal)/aluminum-exposure rat model of AD^38^.

We were able to demonstrate an association of higher levels of GNMPB with increased blood brain barrier disruption as indicated by the GIS in a subset of patients. Of note, a GIS of 0 in a patient with cALD indicates the lack of “active disease”, as defined by insufficient blood brain barrier disruption to result in gadolinium enhancement. One of our four patients with a GIS = 0 had previously documented T2-weighted lesions on MRI. These data suggest the GNMPB may be associated with active demyelination adding to its potential as a biomarker. The presence of a T2 lesion on MRI in this setting indicates that disease was active sometime in the past, or that there is progression but with insufficient inflammation to result in Gad + . These cases are not common and may represent “burned out” or “self-arrested” which have been recently described by Mallack et al.^39^. There may be up to 12% of persons with cALD who have self-arrested^39^. Our subgroup analysis shows these individuals appear to have the lowest levels of GNMPB in their CSF, correlating with a reduced or arrested inflammatory state.

For the first time, we have demonstrated CSF GNMPB correlates with several imaging and clinical aspects of cALD. To become more robust as a biomarker of active demyelination, GNMPB should be studied in prospective trials from boys without cerebral disease longitudinally. Importantly, although our investigations utilized CSF, plasma would be a more ideal body fluid as it is easily accessible in a repeatable manner.

## Methods

### Participants

Samples were obtained from patients with cALD (n = 67, median age of 8.22 years). The patients underwent a lumbar puncture as part of their initial clinical evaluation. Reference CSF was obtained from children (n = 11, median age of 10 years) receiving intrathecal maintenance chemotherapy to treat acute lymphoblastic leukemia (ALL) without cerebral leukemia involvement. Obtaining control samples from patients with scheduled phlebotomy or lumbar puncture reduces the risk that would be presented to “healthy” children. This is the most suitable control, and its use has been previously published^27,28^. This study and the use of all patient samples were approved by the Institutional Review Board at the University of Minnesota (protocol code 0808M42321and date of last approval: 9/30/2021). The study was conducted according to the guidelines of the Declaration of Helsinki. Informed consent was obtained from all subjects involved in the study and/or their parents or guardians, and patient assent was obtained for individuals 8 years of age or older.

### GNMPB measurement

Levels of GNMPB were quantified using the Osteoactivin Human Enzyme-Linked Immunosorbent Assay (ELISA) kits (ab193711, Abcam, Waltham, MA); the samples were run neat.

### Chitotriosidase measurement

Levels of CSF chitotriosidase were measured as previously described^26,40^. In brief blood or CSF samples were diluted in buffer [10 mM Tris–HCL, 15 mM NaCL, pH 7.5], and 20 µl aliquots of these dilutions were incubated with 20 μl of 22 μM 4-methylumbelliferyl-beta-D-N,N’,N’-triacetyl- chitotriose (MUTAC; Sigma, St. Louis, MO; Cat. #M5639) in 0.5 M citrate–phosphate buffer, pH 5.2, in 0.1% Albumin (Sigma, Cat. #A8412) pre-coated 96 well plates (Fisher; Pittsburgh, PA; Cat. #353,072) for 1 h at 37 °C. The reaction was stopped after 1 h with 250 µl 0.5 M Na2CO3-NaHCO3 buffer, pH 10.7. Enzymatic cleavage of MUTAC produces a fluorescent pro- duct, 4-methylumbelliferone (4-MU), which was read on a Molecular Devices, SpectraMAX Gemini fluorometer with 365 nm excitation and 450 nm emissions. The comparison of relative fluorescent units (RFU) with CHIT standards (R&D, Minneapolis, MN; Cat. #3559-GH) ranging from 0.4 to 12.5 ng/well allowed calculation of CHIT activity, which is expressed as nmoles 4-MU generated/mL of sample per hour.

### Statistical methods

Student’s two-tailed t-test was used to evaluate significance, calculated using PRISM. Bivariate fit regression, one-way analysis, and Tukey analysis were performed using PRISM. P-values are included in figures or figure legends.
